# Caspase inhibition improves viability and efficiency of liposomal transfection

**DOI:** 10.1038/s41598-023-49027-y

**Published:** 2023-12-10

**Authors:** Tatsushi Yoshida, Kenta Yamasaki, Kenjiro Tadagaki

**Affiliations:** Department of Biochemistry and Molecular Biology, Graduate School of Medical Science, Kyoto Prefectural University of Medicine, Kawaramachi-Hirokoji, Kamigyo-ku, Kyoto, 602-8566 Japan

**Keywords:** Biological techniques, Molecular biology

## Abstract

High transfection efficiency is the most important point for experiments of DNA and RNA introduction into cells. Decrease of cell viability during the transfection procedure is a crucial issue, resulting in transfection failure. However, the mechanism underlying cell growth inhibition has not been fully elucidated. Lipofection is frequently used for transfection experiments, whereases, depending on cell type, it causes a decrease in cell viability. The present study demonstrates here that a potent pan-caspase inhibitor Q-VD-OPh blocked cell death during the lipofection, indicating apoptosis was induced in lipofection. Moreover, Q-VD-OPh drastically increased transfected cells. This method provides easier and more effective transfection system of lipofection and may be useful for transfection of not only cell lines but also clinical uses such as gene therapy and nucleic acids vaccine.

## Introduction

Transfection is an essential method to elucidate genetic functions. The first transfection method is calcium phosphate -mediated transfection in which DNA-binding calcium phosphate precipitates are introduced into cells^1,2^. Then lipid-mediated DNA transfection method, lipofection, was developed^3,4^. Cationic lipids interact with anionic DNA and the conjugates are used for gene delivery into cells^5,6^. Brief heat shock enhanced the transfection efficiency, but the effects was moderate^7^. Lipofectamine 2000 reagent is currently and widely used for gene delivery procedure^8^, however its transfection rate depends on cell types and cell toxicity of the reagent is a crucial issue. The underlying molecular mechanism of the cell toxicity exerted by lipofection has not been fully elucidated.

Cell death is classified to three major types by the forms and the molecular mechanisms^9,10^. Type I is apoptosis, type II is autophagic cell death and type III is necrotic cell death. In addition, recent studies discovered novel types of cell death such as necroptosis^11,12^, ferroptosis^13^ and pyroptosis^14^.

Apoptosis was a form of cell death reported by pathologist Kerr JF in 1972^15^. It has been named apoptosis, a combination of apo (off) and ptosis (falling), derived from the Greek word meaning to fall away from its appearance under a microscope. There was a group of cell populations that were lost through orderly cell death during organogenesis in living organisms, and the concept of programmed cell death was born^16,17^. It was found that apoptosis occurs in the process of programmed cell death, and the molecular induction mechanism of apoptosis has been elucidated^18–22^. Apoptosis-inducing mechanisms can be broadly divided into extrinsic pathways and intrinsic pathways^23,24^. In the extrinsic pathway, a death ligand secreted on the cell membrane or extracellularly binds to a death receptor that exists as a transmembrane protein on the cell membrane of the target cell and initiates intracellular apoptosis^25,26^. In the intrinsic pathway, stimuli such as DNA damage that induce apoptosis are transmitted to mitochondria through the induction of the expression of gene clusters that respond to the stimuli^27^. The apoptotic stimuli increase the permeability of the mitochondrial outer membrane, resulting in cytochrome C release into the cytoplasm^28,29^. Both extrinsic and intrinsic apoptosis pathways lead to cysteine aspartate-specific protease (caspase) cleavage and activation. Activated caspases cleave a number of substrate proteins, leading to execution of apoptosis. Quinoline-Val-Asp-Difluorophenoxymethylketone (Q-VD-OPh) is a broad-spectrum caspase inhibitor and used as a potent apoptosis inhibitor^30,31^. Q-VD-OPh is used for examination of apoptotic signaling in vitro and in vivo^32^.

Present study here demonstrates that cell toxicity caused by lipofection is apoptosis and the lipofection together with Q-VD-OPh is a novel method to strongly improve the transfection efficiency.

## Materials and methods

### Cell culture

Cervical cancer cell line HeLa was obtained from Riken BioResource Center and Human Pancreas Adenocarcinoma cell line AsPC-1 was obtained from American Type Culture Collection (ATCC). Cells were cultured in Dulbecco’s modified Eagle medium (DMEM) (Fujifilm Wako Pure Chemical Corporation) with 10% Fatal bovine serum (FBS) (Sigma-Aldrich; Merck KGaA) and 2 mmol/l glutamine (Gibco; Thermo Fisher Scientific Inc.). Cells were maintained at 37 °C in humidified air with 5%CO_2_.

### Transfection

Cells were inoculated into 24 well plate at 0.5 × 10^5^ cells/well. After 24 h incubation, pmaxGFP plasmid (Lonza) (1 microg/well) was transfected into cells using 1 microl of lipofectamine 2000 (Thermo Fisher Scientific) as described previously^33^. Caspase inhibitor Q-VD-OPh (MedChemExpress) or Z-VAD-FMK (R&D systems, Minneapolis, MN) was added to cells at 100 microM, 20 min before transfection.

### Cell counts

Forty-eight hours after transfection, cells were detached from the bottom of well by trypsin and harvested. Equal amount of trypan blue solution was mixed with the harvested cells and observed using a Burker-Turk counting chamber (Erma. Inc) under an Olympus CK40 inverted light microscope (Olympus Corporation). Blue cells stained with trypan blue, which could not exclude trypan blue from cytosol to outside of cells, were evaluated as dead cells.

### GFP fluorescence detection

Cells were observed under an inverted microscope (Olympus CK40) with a fluorescent unit U-RFLT50 in a dark room. Photographs were taken by Olympus digital camera CAMEDIA C-5050 zoom.

### Flow cytometry analysis

Harvested cells were incubated with propidium iodide (PI) solution containing PBS, 0.1% triton- × 100 and 10 microg/ml of propidium iodide. PI stained cells were analyzed by BD FACS Canto II and BD FACS diva (BD Biosciences). To detect GFP signal, cells were suspended in PBS and analyzed by BD FACS Canto II and BD FACS diva.

### Western blotting

Cells were suspended in RIPA buffer and centrifugated. Supernatant was collected as cell lysate. Proteins of the cell lysate were resolved in 7.5% sodium dodecyl sulfate–polyacrylamide gel (SDS-PAGE) and proteins were transferred to a nitrocellulose filter. Proteins on the filter were reacted with poly (ADP-ribose) polymerase (PARP) antibody (#9542) (Cell signaling technology, Danvers, MA) or β-actin antibody (PM053) (Medical and Biological Laboratories, Tokyo, Japan). The signals were detected by ECL Western blotting detection reagents (GE healthcare, Chicago, IL) and image quant LAS500 (GE healthcare, Chicago, IL).

### Statistical analysis

Data were statistically analyzed using a two-tailed, unpaired Student’s t-test by Microsoft Excel (Microsoft Corporation).

## Results

### Q-VD-OPh increased GFP-positive cells during lipofection.

**Figure 1:**
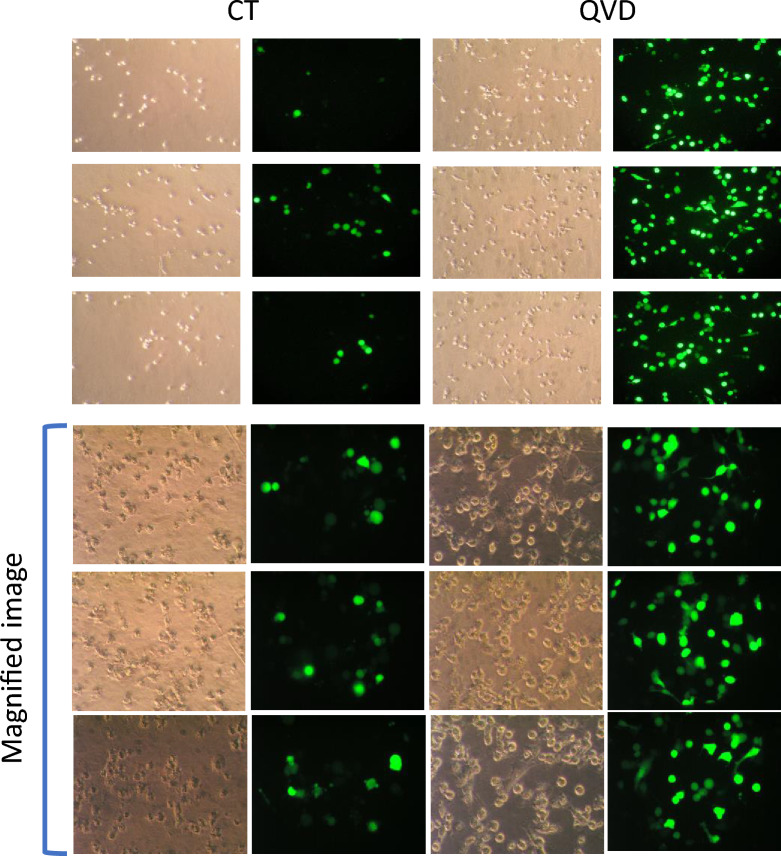
Q-VD-OPh increased GFP-positive cells during lipofection. HeLa cells were transfected with a plasmid carrying GFP gene (pmaxGFP) by lipofection method and observed under a microscope. Caspase inhibitor Q-VD-OPh were added at 100 microM before the transfection. Upper panels, magnification ×100. Lower panels, magnification ×200. *CT* control without Q-VD-OPh treatment. Observed views of different three culture wells are shown.

Cervical cancer cell line HeLa cells are frequently used for molecular biological studies but the transfection rate is not fragrant by lipofection. As shown in Fig. 1, a little of cells gave off green light when plasmid carrying GFP gene was transfected by lipofection (Left panels shown as CT) and GFP signals on the bottom of the culture well were observed. Some cells were detached from the bottom of culture dishes and floated, resulting in low transfection efficiency. Q-VD-OPh is a potent pan-caspase inhibitor and can block apoptosis induction^30,31^. Addition of Q-VD-OPh increased the number of cells with GFP’s green light (Right panels shown as QVD).

### Apoptosis was increased by lipofection

**Figure 2:**
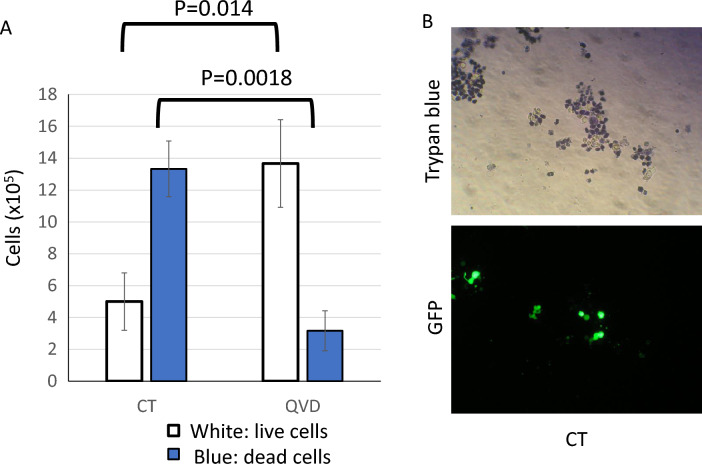
Apoptosis was increased by lipofection. Cell viability after transfection was measured by trypan blue dye exclusion assay. (**A**) Only trypan blue assay was performed and cells were counted. (**B**) GFP observation was simultaneously done with trypan blue assay. Cells stained with blue were evaluated as dead cells. Cells stained without blue were observed with white and evaluated as live cells. The data was n = 3 ± S.D.

To examine whether cells died by transfection damage, trypan blue dye exclusion assay was performed. In the examination, all cells including floated cells of the culture well were collected and analyzed. More than a half of cells were stained with blue without Q-VD-OPh (Fig. 2A), meaning that the transfection damage caused dead of cells. Q-VD-OPh conversely increased live cells with white, indicating that the apoptosis was induced by the transfection damage. We simultaneously performed trypan blue dye exclusion assay and GFP observation (Fig. 2B). Dead cells did not show green fluorescence but a part of live cells showed GFP signals.

### Q-VD-OPh blocked apoptosis but not affected cell cycle during lipofection

**Figure 3:**
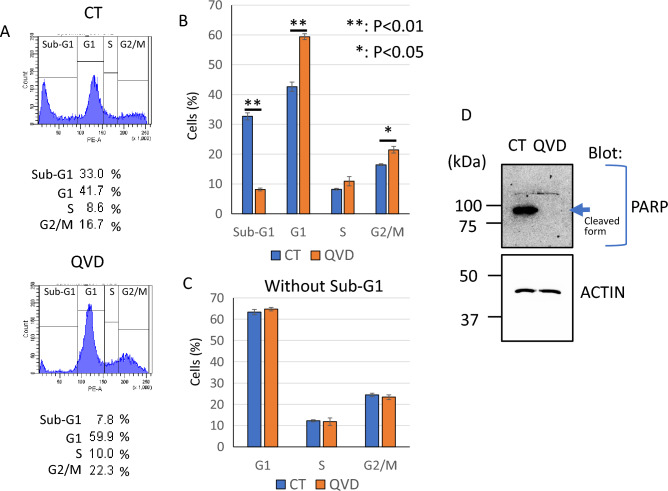
Q-VD-OPh blocked apoptosis but not affected cell cycle during lipofection. Cell cycle analysis was performed by flowcytometry. (**A**) Representative histograms. (**B**) Bar graph of cell cycle analysis. (**C**) Bar graph of cell cycle analysis except for sub-G1 populations. The data was n = 3 ± S.D. (**D**) Western blotting of PARP and ACTIN. *CT* control without Q-VD-OPh treatment.

Next, the affect of transfection damage on cell cycle was examined by flowcytometry. As shown in Fig. 3A, B, transfected cells showed more than 30% of sub-G1population. On the other hand, sub-G1 of Q-VD-OPh treated cells was less than 10% and G1and G2/M populations were increased compared to transfected cells without Q-VD-OPh. When G1, S and G2/M were calculated except for sub-G1, these are almost the same between with Q-VD-OPh and without Q-VD-OPh (Fig. 3C). The result indicated that when dead cells were eliminated, cell cycle populations of remaining cells were not affected by Q-VD-OPh. In addition, to confirmed apoptosis induction, we analyzed PARP cleavage by western blotting. PARP is a substrate of caspases. When GFP plasmid was transfected into HeLa cells, cleaved PARP was detected and Q-VD-OPh blocked the cleavage (Fig. 3D).

### Q-VD-OPh improved transcription rate of lipofection

**Figure 4:**
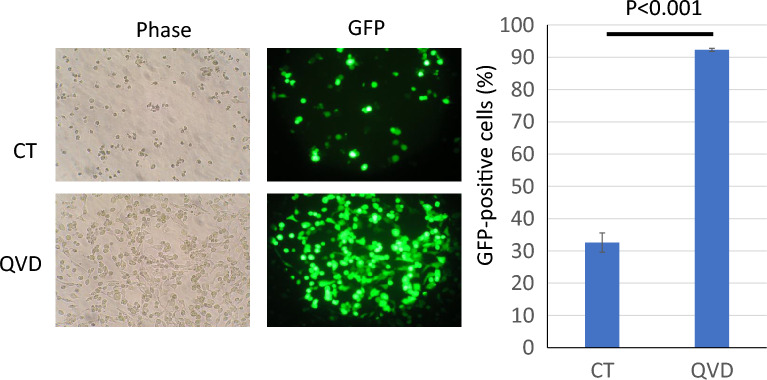
Q-VD-OPh improved transcription rate of lipofection. The rate of GFP positive cells against total cells was calculated and shown as a bar graph. The data was n = 3 ± S.D. *CT* control without Q-VD-OPh treatment.

**Figure 5:**
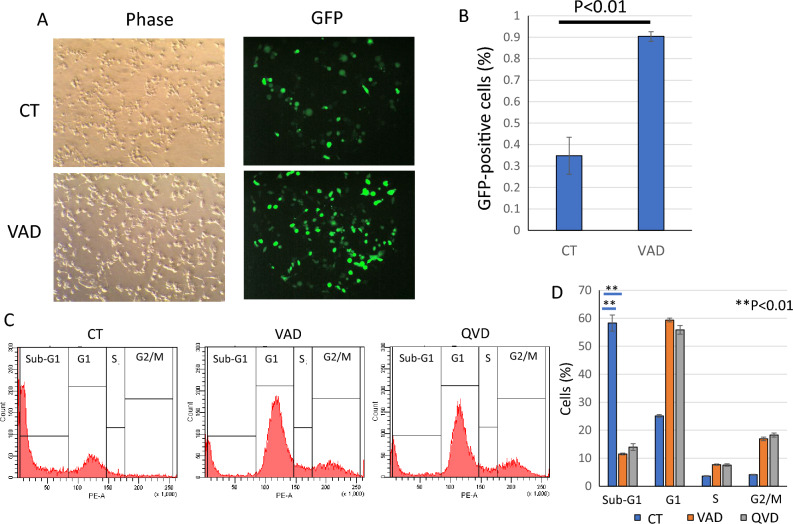
Z-VAD-FMK improved transfection rate and viability of lipofection. The rate of GFP positive cells against total cells was calculated. Observed images by a microscope (**A**) and a bar graph (**B**) were shown. Cell cycle analysis was performed by flowcytometry. (**C**) Representative histograms. (**D**) Bar graph of cell cycle analysis. The data was n = 3 ± S.D. *CT* control without Q-VD-OPh nor Z-VAD-FMK treatment.

**Figure 6:**
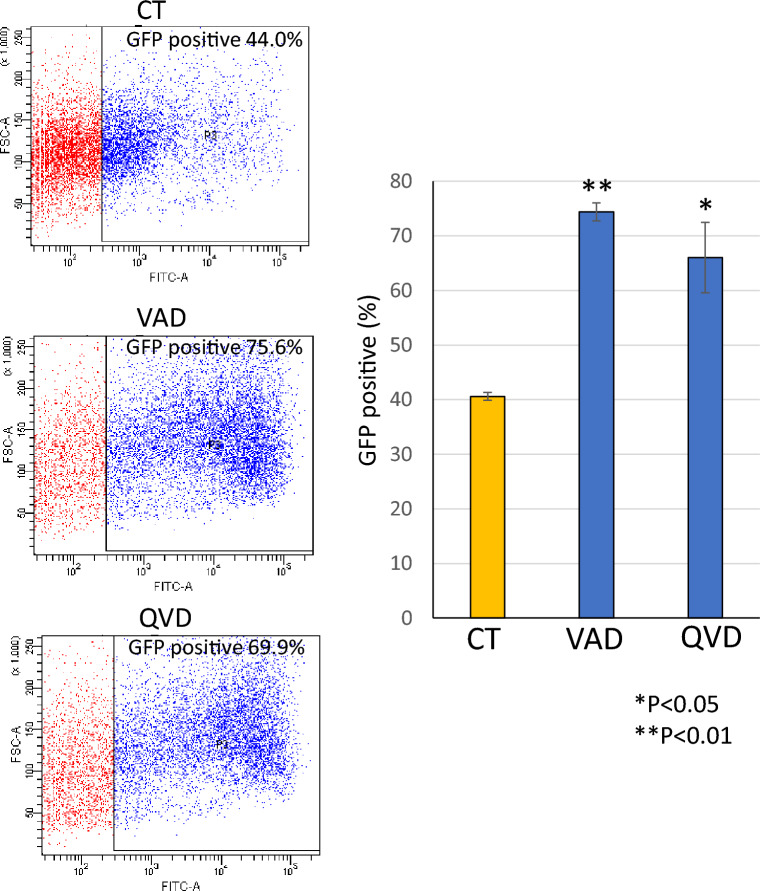
Caspase inhibitors improved transfection rate of lipofection. The rate of GFP positive cells was detected by flow cytometry. Representative FACS data and a bar graph were shown. The data was n = 3 ± S.D. *CT* control without Q-VD-OPh nor Z-VAD-FMK treatment.

Bright-field image and fluorescent image of GFP were observed and transfection rate was evaluated (Fig. 4). Marged cells with green fluorescence were counted and the rate against all cells was shown in a bar graph. Compared to the rate of cells without Q-VD-OPh (about 30%), most of Q-VD-OPh treated cells showed green fluorescence and the transfection rate was increased to more than 90%. Next, another caspase inhibitor Z-Val-Ala-Asp(OMe)-CH2F (Z-VAD-FMK) was used. Z-VAD-FMK also increased the number of transfected cells and blocked cell death induced by liposomal transfection (Fig. 5). As an alternative method, flow cytometry was used to analyze rate of GFP positive cells. The results also showed that caspase inhibition by Q-VD-OPh or Z-VAD-FMK increased the rate of GFP positive cells (Fig. 6). In not only HeLa cells but also Human Pancreas Adenocarcinoma cell line AsPC-1, these inhibitors blocked cytotoxicity and increased rate of transfected cells in liposomal transfection (Supplementary Data 1).

### The viability and expression of transfected plasmid continued after Q-VD-OPh removal

**Figure 7:**
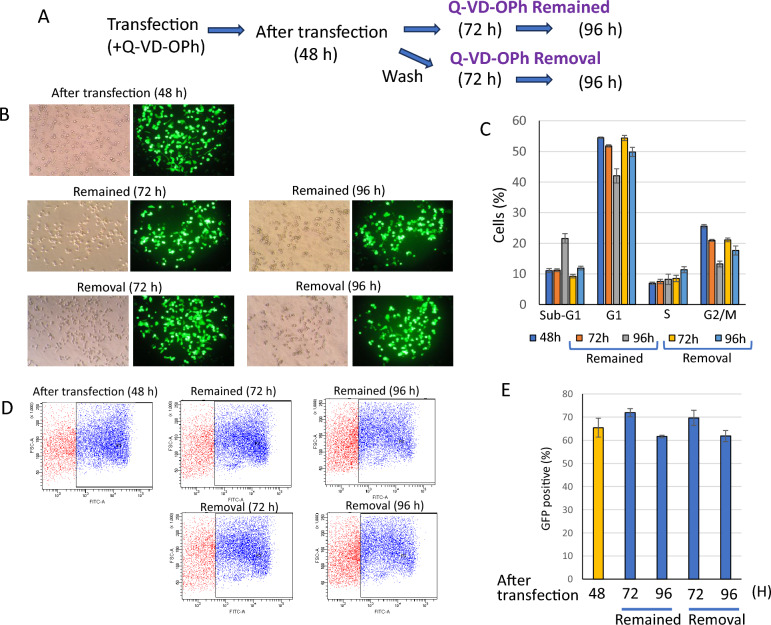
The viability and expression of transfected plasmid continued after Q-VD-OPh removal. (**A**) Schematic diagram of the experiment. (**B**) Observed images by a microscope. (**C**) Bar graph of cell cycle analysis. (**D,E**) Rate of GFP positive cells detected by flow cytometry. Representative FACS data (**D**) and a bar graph (**E**) were shown. The data was n = 3 ± S.D.

Caspase inhibitors increased the cell viability and the rate of liposomal transfection, however a question remained whether the effect would continue even after Q-VD-OPh was removed. We conducted an experiment to examine the effects after drug removal. Forty-eight hours after transfection, culture medium containing Q-VD-OPh was replased to fresh medium without Q-VD-OPh following washing of cells with medium twice. We examined viability and GFP expression at 72 or 96 h after transfection. Cells after Q-VD-OPh removal survived and expressed GFP to the same extent as cells in which Q-VD-OPh remained (Fig. 7). These results indicated that once transfection damage was escaped, cells could keep alive and expression even when caspase inhibitor was removed.

## Discussion

Cell toxicity was a crucial issue of gene transfer procedure by lipofection and the present study demonstrated here the method to solve the severe problem. The results indicate that once transient cell damage is blocked, the rate of gene introduction into cells is highly enhanced. The method is useful for analysis of genetic functions and production of large amount of recombinant proteins but a precaution is needed in the studies involved in signaling pathway and protein function especially in the aspect of apoptosis. A potent pan-caspase inhibitor Q-VD-OPh completely blocked the cell death during lipofection, which means lipofection executed caspase-dependent apoptosis among some types of cell death. Previous studies have reported the relationship between transfection and apoptosis. Electrotransfection induced apoptosis and cell viability and electrotransfection efficiency of lymphoma cells were improved by a treatment of caspase inhibitor^34,35^. Infection of influenza virus executed apoptosis and caspase inhibition or caspase 3 knockdown impaired virus propagation^36^. Lipofection caused apoptosis in lymphoma or lymphocyte^34,37^ and tumor-necrosis factor (TNF) secretion mediated the apoptosis^37^, though the effect of caspase inhibitor was not examined.

Interestingly, the caspase inhibition by Q-VD-OPh increased the transfection rate as well as the cell viability. The result suggested that caspases blocked the introduction of exogenous plasmid DNA into cells or gene expression of the introduced plasmid DNA. Activated caspases can activate deoxyribonuclease (DNase) such as DNase II^38^ and caspase activated DNase (CAD)^39^. Theses DNases may degrade the transfected plasmid DNA. Caspases are suggested to play a role in an innate immune response. Because when DNase II knock downed drosophila by siRNA was infected with Gram negative or positive bacteria, the viability of the flies was severely reduced^40^. Coronavirus disease 2019 (COVID-19) vaccine is lipid-nanoparticle-formulated, nucleoside-modified mRNA vaccine^41–43^. In the future, a lot of nucleic acids vaccines transduced by lipofection will be developed. Thus, lipofection plus Q-VD-OPh may be powerful strategy for nucleic acid vaccination and gene therapy, because it can increase transfection efficiency and reduce cell death at the vaccination and gene transferred part, leading to the decrease of side effects.

The present study demonstrates here a novel and effective method for gene transfer using lipofection and it will be widely used in laboratory and used clinically.

### Supplementary Information

Supplementary Figure 1.

Supplementary Legends.
