# Supplementary figures and images for: Caspase inhibition improves viability and efficiency of liposomal transfection
Source: Sci Rep. 2023 Dec 10;13:21868. doi: 10.1038/s41598-023-49027-y (PMC10711006; doi:10.1038/s41598-023-49027-y)

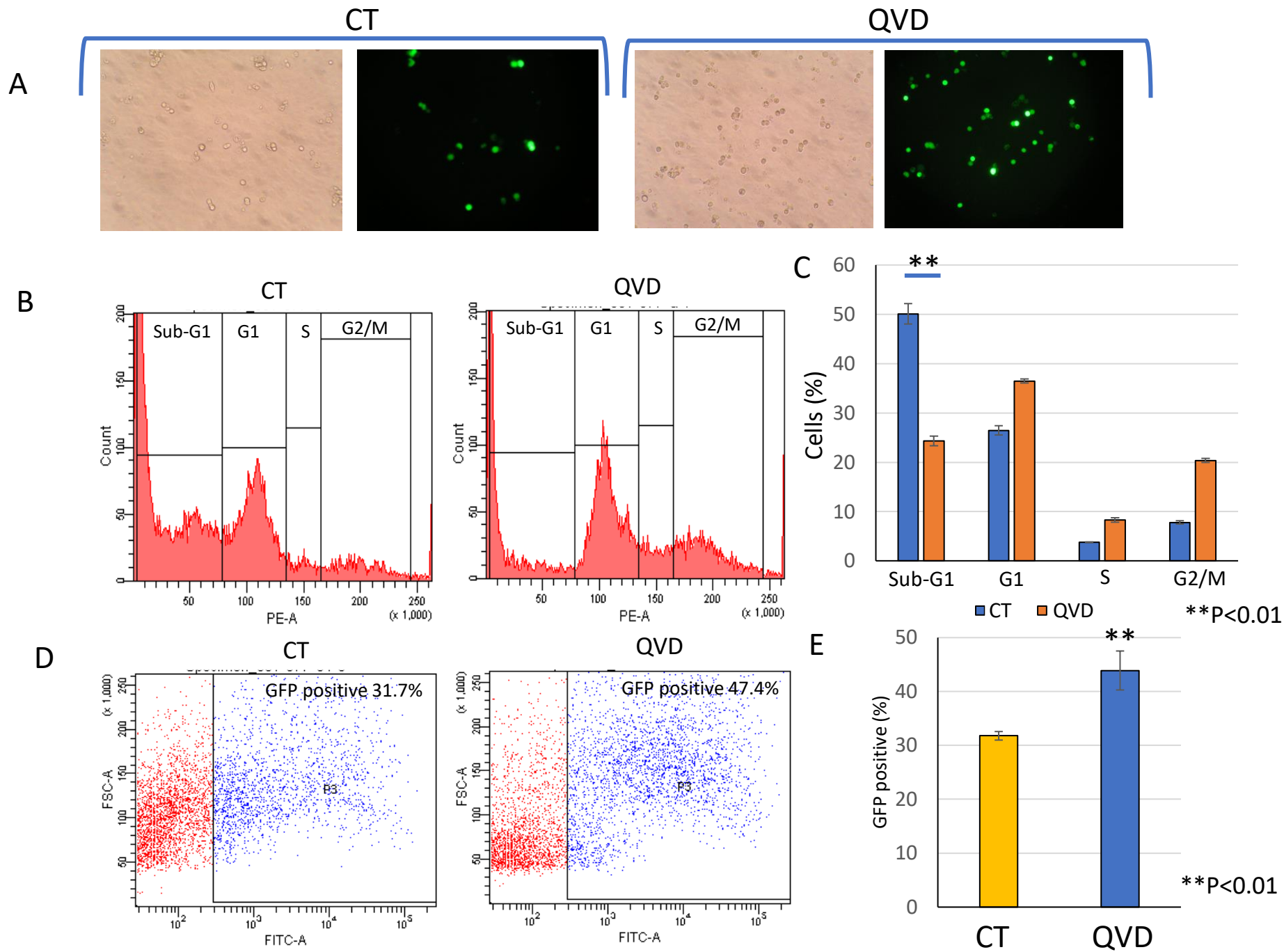

Supplement: Supplementary file 1 — Supplementary Figure 1. [file 41598_2023_49027_MOESM1_ESM.pdf]
