# Supplementary material for: Caspase inhibition improves viability and efficiency of liposomal transfection
Source: Sci Rep. 2023 Dec 10;13:21868. doi: 10.1038/s41598-023-49027-y (PMC10711006; doi:10.1038/s41598-023-49027-y)
Supplement: Supplementary file 2 — Supplementary Legends. [file 41598_2023_49027_MOESM2_ESM.docx]

**Supplemental data 1. Q-VD-OPh improved viability and transcription rate of lipofection in AsPC-1 cells.**

(A) Observed images under a microscope. (B and C) Cell cycle analysis. Representative histograms (B) and a bar graph (C) were shown. (D and E) The rate of GFP positive cells was detected by flow cytometry. Representative FACS data (D) and a bar graph (E) were shown. The data was n=3+/-S.D.. CT, control without Q-VD-OPh treatment.
